# The Threshold of the Severity of Diabetic Retinopathy below Which Intensive Glycemic Control Is Beneficial in Diabetic Patients: Estimation Using Data from Large Randomized Clinical Trials

**DOI:** 10.1155/2020/8765139

**Published:** 2020-01-16

**Authors:** Yuqi Liu, Juan Li, Jinfang Ma, Nanwei Tong

**Affiliations:** Department of Endocrinology and Metabolism, West China Hospital of Sichuan University, Chengdu, 610041 Sichuan, China

## Abstract

Intensive glucose therapy can protect the retina of individuals with diabetes, but it is unknown if it provides the same protection to patients with different severity of diabetic retinopathy (DR). We finally included DR-related studies involving intensive glucose control with large sample size and long follow-up time, including five large and high-quality randomized clinical trials (RCTs): DCCT, UKPDS, ACCORD, AdRem, and VADT. With DCCT as a reference, we supposed a DR severity threshold that is verified by other RCTs then. We found that individuals who have DR lesions that are equivalent to or less severe than moderate NPDR achieve benefits for the retina by intensive glycemic control. However, these are realized only if the HbA1c in type 1 or type 2 diabetic patients is reduced at least by 0.8% versus the control group or it is reduced to <7% and >3 years of intensive glucose control is required. If the severity of DR lesions is worse than moderate NPDR, intensive glycemic control may not bring benefits.

## 1. Introduction

Diabetic retinopathy (DR) is a progressive disease that can be divided into two stages: the earlier stage is referred to as “nonproliferative diabetic retinopathy” (NPDR) and the later stage as “proliferative diabetic retinopathy” (PDR). NPDR is characterized by weakness of the capillary wall, microaneurysm formation and fluid leakage, and greater adhesion of leukocytes and monocytes to the endothelium [1]. Conversely, the proliferative stage is characterized by the development of new retinal blood vessels and fibrous tissue at the optic disc or near venules elsewhere in the retina. The appearance of these vessels is associated with nonperfusion or edema in the macula, vitreous hemorrhage, and distortions or traction retinal detachment, leading to loss of vision [2].

Studies of intensive glycemic control in patients with newly diagnosed type 1 diabetes and type 2 diabetes, including the Diabetes Control and Complications Trials (DCCT) [3] and the United Kingdom Prospective Diabetes Study (UKPDS), and their follow-up studies [4, 5], showed a legacy effect of intensive glucose control in terms of macrovascular protection in those patients without established atherosclerotic cardiovascular disease (ASCVD). However, a previously published meta-analysis found that intensive glucose control does not have such a legacy effect in diabetic patients with established or very high-risk ASCVD [6]. The current evidence suggests that intensive glycemic control can reduce the incidence of diabetic retinopathy and delay the progression of retinopathy in patients with type 1 or type 2 diabetes in the DCCT [3], its follow-up study [7], and UKPDS [4], but there is few focused on whether disparate effects exist in individuals with different DR conditions.

The retinal microvascular protection provided by intensive glucose therapy may depend on the severity of the lesions. We hypothesized that there is a particular threshold of lesion severity below which hypoglycemic therapy, especially intensive glycemic control or glycemia-dependent retinal microvascular protection, can be effective. Conversely, once the lesion reaches a certain degree of severity, intensive glycemic control will not have microvascular benefits. In this review, we aim to infer the threshold of DR lesion severity below which intensive therapy could have protective effects, but above which it would have no benefits for patients. We have pursued this aim by reviewing high-quality published randomized clinical trials (RCTs) of intensive blood glucose control in diabetic patients.

## 2. Search Strategy and Selection Criteria

We screened published literature searched according to the search strategy (Supplement [Supplementary-material supplementary-material-1]). Randomized controlled trials were included if they separately assessed the effects of intensive glycemic control in individuals with type 1 or type 2 diabetes and diabetic retinopathy has been reported and if they had at least a 3-year follow-up in both groups and had more than 500 participant-years in each treatment group. The intensive glycemic control has been defined as maintenance of glycated hemoglobin A1c (HbA1c) concentration at ≤7% (53 mmol/mol) in the intervention group or a difference in HbA1c between the intervention and conventional management groups of ≥0.8% [8]. Trials were excluded if they were in a non-English publication or with multifactorial interventions that cannot separate assessment of the effects of glycemic control.

The primary outcomes were new-onset or any progression in diabetic retinopathy which was defined as a composite of progression of DR by at least two steps on the Early Treatment of Diabetic Retinopathy Study (ETDRS) severity scale, development of proliferative retinopathy, or requirement for retinal photocoagulation therapy or vitrectomy.

## 3. Determination of the DR Severity Threshold below Which Intensive Glycemic Control Has Benefits for the Retina

### 3.1. Homogeneity of Assessment of DR Severity in RCTs

Finally, five trials were included in this review (Table 1). Although each study was different, the description and classification of DR were based on the Early Treatment of Diabetic Retinopathy Study (ETDRS) [9]. Based on data from the ETDRS and the Wisconsin Epidemiological Study on Diabetic Retinopathy (WESDR) [10], the Retinopathy Disease Severity Scale (DR International Classification Standard, Table 2) and the International Clinical Diabetic Macular Edema Disease Severity Scale were developed in Sydney in 2002 by the American Academy of Ophthalmology (AAO), involving representatives from many countries. The standard scale provides an important basis for standardizing epidemiological investigations of DR, facilitates communication among community doctors, endocrinologists, and ophthalmologists, and is widely applied internationally [11]. The UKPDS [4] made minor adjustments to the ETDRS grading scale (Table 3), but this did not affect the uniformity of the severity scales (Table 2). The methods of fundic assessment and grading in studies we included were almost identical to those of the UKPDS.

In summary, the International Clinical DR Disease Severity Scale can be used to assess the severity of DR in subjects in the studies we included, and the criteria used can be regarded as consistent.

In these studies, the progression of DR was defined as the increase in severity of DR from baseline at follow-up of ≥2 or ≥3 steps in the ETDRS grading system, where an increase of 1 step means that the severity of the lesion has progressed from the original level to the next more severe level. Analysis of the ACCORD study showed that the definition of progression used (>1, 2, or 3 steps) does not affect the results of the study, so it can be considered that the definition of DR progression is similar for each study [12].

### 3.2. Identification of the Hypothesized DR Severity Threshold

Because DR is a classical and specific complication, the severity of which is closely related to blood glucose concentration in type 1 diabetes, we first decided to identify such a threshold using data from the DCCT. The DCCT study, initiated in 1983, was an RCT conducted in patients with type 1 diabetes. The study was of 1,441 participants and included a primary prevention cohort of 726 patients who had no retinopathy and a secondary intervention cohort of 200 individuals with microangioma or nonproliferative DR in 715 participants [3].

The secondary intervention cohort excluded patients with severe NPDR and those with more severe DR, meaning that the fundic status of all the subjects in this group was at or below the moderate NPDR level of the International Clinical DR Disease Severity Scale. The mean HbA1c at baseline was 8.8%, and the mean follow-up duration was 6.4 years. At the end of the study, the mean HbA1c concentrations achieved were 7.2% and 8.0% in the intensive glycemic control and conventional management groups, respectively. The endpoints were the incidence of DR and progression of DR, with the progression of DR being defined as a severity increment of ≥3 steps in the ETDRS grading system between baseline and follow-up.

In the primary prevention cohort of the study, there was a significant difference in the cumulative incidence of DR between the two groups at 36 months, which is why we only included RCTs with more than a 3-year follow-up. From 5 years onward, the cumulative incidence of retinopathy in the intensive therapy group was approximately 50% less than that in the conventional treatment group, and after a mean 6-year follow-up, intensive glucose control reduced the adjusted mean risk of retinopathy by 76%. The reduction in risk increased with time [3].

The patients in the intensive glycemic control group in the secondary intervention cohort had a higher cumulative incidence of progression of DR. Intensive treatment reduced the mean risk of DR progression by 54% over the entire study period (*n* = 77 events in the intensive group and *n* = 143 events in the conventional group). It appears that intensive glycemic control can delay the progression of DR in patients whose lesion severity is less than or equal to the moderate NPDR on the International Clinical DR Disease Severity Scale. Therefore, we contend that the DR severity threshold below which significant retinal benefits of intensive glucose control accrue is no more than that consistent with moderate NPDR, as defined in the International Clinical DR Disease Severity Scale.

### 3.3. Verification of the Determined DR Severity Threshold

The UKPDS included 3,867 newly diagnosed type 2 diabetes patients with a median age of 54 years, 64% of whom had no NPDR at the time of admission, 24% had mild NPDR, 10% had moderate NPDR, 2% had severe NPDR, and 0.1% had PDR, meaning that >95% of the participants had no DR, or their DR was less severe or equivalent to moderate NPDR at the beginning of the study [4, 13]. After 10 years of follow-up, the HbA1c concentration achieved in the intensive glycemic control group was 7.0%, compared with 7.9% in the conventional therapy group. At the commencement of the study and then every 3 years, all the participants underwent a full clinical examination, including tests of visual acuity and ophthalmoscopy following pupillary dilation. There was a significant difference in the incidence of progression of DR between the groups from the sixth year onwards. The results suggested that intensive glycemic control slowed the progression of DR, and it is also consistent with intensive blood glucose control having a beneficial effect on individuals with diabetes when their severity of DR does not exceed the moderate NPDR level defined by the International Clinical DR Disease Severity Scale. However, the study did not report the incidence of newly diagnosed DR.

In the ACCORD study, the effect of intensive glycemic control was evaluated on the incidence of cardiovascular events in type 2 diabetes patients with established cardiovascular disease and/or additional cardiovascular risk factors [14]. A total of 2,856 patients were enrolled, and 38% had at least one cardiovascular event or possessed other CVD risk factors. According to the International Clinical DR Disease Severity Scale, less than 2% had severe NPDR or PDR. The median follow-up period for the study was 4 years, during which the mean HbA1c concentration decreased from >8% to 6.3% in the intensive glycemic control group, but only to 7.6% in the conventional therapy group. The cumulative incidence of progression of DR over the 4 years differed significantly between groups (4.8% in the intensive treatment and 13.1% in the conventional management group) [14]. DR progression was significantly reduced by intensive glycemic intervention compared to standard therapy throughout the study. For all the DR severity levels combined, the primary results showed a statistically significant benefit of intensive glycemic control compared with conventional management [14]. Chew et al. considered specific components of the primary eye outcomes from ACCORD and compared the results among subgroups of different DR severity at baseline [12]. The difference was large and statistically significant only for patients with microaneurysms in one or both eyes when compared with those with mild NPDR in only one eye (odds ratio 0.30, 95% confidence interval 0.15–0.59; *P* = 0.0002), and similar results were obtained for retinopathy progression by 1, 2, and 4 or more steps on the person scale and for ≥2 steps on the eye scale [12]. Although there was no significant difference between the intensive glycemic control group and the control group if progression was defined as >4 steps, this may be because few patients progressed this much, meaning that the analysis was insufficiently powered or that most patients whose DR progressed >4 steps had undergone further photocoagulation. However, the ACCORD trial did show that intensive glycemic control delays the progression of DR, especially in patients with unilateral or bilateral microaneurysms or those with mild NPDR signs in one eye.

These two studies confirmed the beneficial effects of intensive glucose control in the population they enrolled in.

The Action in Diabetes and Vascular Disease: Preterax and Diamicron MR Controlled Evaluation Retinal Measurements (AdRem) study is a substudy of ADVANCE. Of these, over 50% of participants had no DR at baseline, more than 30% had moderate NPDR, and 3.7% had severe NPDR or PDR, which means more participants with severe DR compared with the population in UKPDS and ACCORD. The results indicated that there was no statistical difference in the incidence or frequency of progression of DR between the intensive glycemic control and conventional management groups. The reason for this negative result is unclear, but the worse DR condition at baseline might partly contribute. Besides, although intensive glucose control achieved a median HbA1c of <7%, the difference in the medians between the groups was <0.8% (6.4% *vs*. 7.0%), which might be the explanation because the protective effect of glucose lowering against macrovascular disease was previously shown to require the difference in achieved HbA1c to be ≥0.8% [15].

The VADT [16, 17] study assessed 858 patients with type 2 diabetes using the ETDRS grading system; using the international clinical DR severity scale, 31% of the patients showed no evidence of fundic lesions at the time of enrollment, 21% had mild NPDR, 42% had moderate NPDR, and around 6% had severe NPDR or PDR. Individuals with severe DR were more. The VADT study group defined the progression of DR as a progression of >2 steps between baseline and follow-up. The results of a 5-year follow-up showed that HbA1c in the intensive glycemic control group had decreased from 9.3% to 6.9% on average, while HbA1c in the control group only decreased from 9.4% to 8.4%. Although the two-step progression in the intensive glycemic control group was significantly lower than that in the conventional management group (17.0% *vs*. 22.1%) [16], the other DR outcomes show no significant difference between the two groups. DR condition at the baseline might be a clue for the negative results.

## 4. Discussion

We reviewed five large-scale RCTs which had shown that a DR severity of no more than moderate NPDR on the International Clinical DR Disease Severity Scale is the threshold below which intensive glucose control has beneficial effects on retinal microvessels.

When individuals have DR lesions that are equivalent to or less severe than moderate NPDR, >3 years of intensive glucose control is required to achieve benefits for the retina. However, these are realized only if the HbA1c in type 1 or type 2 diabetic patients is reduced at least by 0.8% versus the control group or it is reduced to <7%. The same degree of glucose control is associated with less pronounced retinal benefits in type 2 diabetic patients than in those with type 1 diabetes, according to analysis of the DCCT, UKPDS, and ACCORD, which might be the result of the presence of hypertension and/or other risk factors frequently associated with type 2 diabetes, such as hypertriglyceridemia.

The increase of permeability in retinal vessels is one of the early pathological signs of DR in animal models. Hyperglycemia induces intracellular reactive oxygen species (ROS) [18] and advanced glycation end product (AGEs) pathway which causes a breakdown of the blood-retina barrier and loss of retinal vascular pericytes [19, 20]. However, overproduction of ROS and decreased efficiency of antioxidant defenses eventually worsen over the course of the disease [21]. This might partly explain why glycemic control lost the protective effect for an individual's retina with severe DR.

It is widely accepted that glycemic control does delay the progression of diabetic retinopathy. However, nearly no guideline or review mentioned whether the different severity of diabetic retinopathy conditions affects the beneficial effect of glycemic control. Based on the benefit-risk evaluation, it is vital for clinicians to make their clinical decisions on appropriate glycemic control goals with their different patients.

We have to admit the inevitable limitation on this review. Because these data have no details in the events in each level of DR severity, a meta-analysis cannot be performed. However, these trials we included can be considered as the “milestones” in diabetes mellitus fields. We can just consider each study as a whole body and infer to find its value.

In summary, we contend that the concept of a threshold of DR severity can provide clinicians with a reference to judge whether the retinas of individuals with diabetes could be protected by intensive glucose control, and it is also a new point that need more attention for future investigations.

## Figures and Tables

**Table 1:** Characteristics of included studies.

Study	Author and year	Country	Age (years) (IG vs. CG)	DM type	Follow-up (years)	HbA1c at baseline (IG vs. CG)	Achieved HbA1c (IG vs. CG)
DCCT	DCCT group, 1993	USA	27	T1DM	10	Primary prevention: 8.8%; secondary prevention: 8.9%	7.2% vs. 8.0%
UKPDS	UKPDS group, 1998	UK	63	T2DM	15	9.3% vs. 9.4%	7.1% vs. 7.9%
VADT	Duckworth 2009, Azad 2014	USA	60	T2DM	6	9.3% vs. 9.4%	6.9% vs. 8.4%
ACCORD	ACCORD group, 2010	USA	61.6	T2DM	4	8.2% vs. 8.2%	7.1% vs. 9.4%
AdRem	Beulens, 2009	20 countries	66	T2DM	4.1	7.4% vs. 7.4%	6.49% vs. 7.24%

DM = diabetes; T1DM = type 1 diabetes; T2DM = type 2 diabetes; IG = intensive glycemia control group; CG = conventional glycemic control group.

**Table 2:** The Diabetic Retinopathy Disease Severity and International Clinical Diabetic Retinopathy Disease Severity Scales.

Disease severity level	Findings observable upon dilated ophthalmoscopy
No apparent retinopathy	No abnormalities
Mild NPDR	Microaneurysms only
Moderate NPDR	More than just microaneurysms but less than severe NPDR
Severe NPDR	
U.S. definition	Any of the following (4-2-1 rule) and no signs of proliferative retinopathy: (i) Severe intraretinal hemorrhages and microaneurysms in each of four quadrants (ii) Definite venous beading in two or more quadrants (iii) Prominent IRMA in one or more quadrants
International definition	Any of the following and no signs of proliferative retinopathy: (i) More than 20 intraretinal hemorrhages in each of four quadrants (ii) Definite venous beading in two or more quadrants (iii) Prominent IRMA in one or more quadrants
PDR	One or both of the following: (i) Neovascularization (ii) Vitreous/preretinal hemorrhage

IRMA = intraretinal microvascular abnormalities; NPDR = nonproliferative diabetic retinopathy; PDR = proliferative diabetic retinopathy. Note: any patient with two or more of the characteristics of severe NPDR is considered to have very severe NPDR. PDR may be classified as high risk or not high risk.

**Table 3:** The grading system from the Early Treatment of Diabetic Retinopathy Study.

Level	Severity	Definitions	Scale step
10/10	DR absent	All diabetic retinopathy features absent	1
20/<20	MA only	Microaneurysm(s) only, other lesions absent, one eye	2
20/20	MA only	Microaneurysm(s) only, other lesions absent, both eyes	3
35/<35	Mild NPDR	MA plus retinal hemorrhage(s) and/or hard exudates and/or cotton wool spots, one eye	4
35/35	Mild NPDR	MA plus retinal hemorrhage(s) and/or hard exudates and/or cotton wool spots, both eyes	5
43/<43	Moderate NPDR	Lesions as above + either extensive or severe HMA or IRMA present, one eye	6
43/43	Moderate NPDR	Lesions as above + either extensive or severe HMA or IRMA present, both eyes	7
47/<47	Moderately severe NPDR	Lesions of 35 + either extensive or severe HMA with IRMA or venous beading, one eye	8
47/47	Moderately severe NPDR	Lesions of 35 + either extensive or severe HMA with IRMA or venous beading, both eyes	9
53/<53	Severe NPDR	Extensive and severe HMA, IRMA, and/or venous beading, one eye	10
53/53	Severe NPDR	Extensive and severe HMA, IRMA, and/or venous beading, both eyes	11
60, 61, 65, 71, 75, 81	Proliferative DR	NVD and/or NVE without or with complication	12+

DR = diabetic retinopathy; NPDR = nonprotective diabetic retinopathy; MA = microaneurysm; HMA = hemorrhages and microaneurysms; HE = hard exudates; CWS = cotton wool spots; IRMA = intraretinal microvascular abnormalities; NVD = new vessels on the disc; NVE = new vessels elsewhere.

## Supplementary Materials

Supplementary Materials Supplement Table 1. Search strategy, Supplement Table 2. The baseline of diabetic retinopathy condition, Supplement Table 3. Diabetic retinopathy outcomes at the end of studies
