# Corrigendum to “The Threshold of the Severity of Diabetic Retinopathy below Which Intensive Glycemic Control Is Beneficial in Diabetic Patients: Estimation Using Data from Large Randomized Clinical Trials” by Yuqi Liu,Juan Li, Jinfang Ma, Nanwei Tong

**DOI:** 10.1155/2020/5498528

**Published:** 2020-06-25

**Authors:** Yuqi Liu, Juan Li, Jinfang Ma, Nanwei Tong

**Affiliations:** Department of Endocrinology and Metabolism, West China Hospital of Sichuan University, Chengdu, 610041 Sichuan, China

In the article titled “The Threshold of the Severity of Diabetic Retinopathy below Which Intensive Glycemic Control Is Beneficial in Diabetic Patients: Estimation Using Data from Large Randomized Clinical Trials” [1], there was an error in the Acknowledgments section, where Grant No. 0040205301D50 should be corrected to Grant No. 2015SZ0228.
