# Supplementary material for: The Threshold of the Severity of Diabetic Retinopathy below Which Intensive Glycemic Control Is Beneficial in Diabetic Patients: Estimation Using Data from Large Randomized Clinical Trials
Source: J Diabetes Res. 2020 Jan 16;2020:8765139. doi: 10.1155/2020/8765139 (PMC6988671; doi:10.1155/2020/8765139)
Supplement: Supplementary Materials — Supplement Table 1. Search strategy, Supplement Table 2. The baseline of diabetic retinopathy condition, Supplement Table 3. Diabetic retinopathy outcomes at the end of studies [file 8765139.f1.docx]

**Supplementary Material**

**Supplement Table 1. Search strategy**

| **Database** | **Search strategy** | **Results** |
| --- | --- | --- |
| Pubmed | ((((diabetes) AND (((tight) OR intensive) OR conventional)) AND (((((((((blood glucose) OR blood sugar) OR glycemia) OR glycaemia) OR glycemic) OR glycaemic) OR glycated hemoglobin A) OR HbA1c) OR A1c)) AND ((diabetic retinopathy) OR retinopath*)) AND ((randomized controlled trial) OR random*) | 196 |
| Embase | ('glucose blood level' OR 'glycosylated hemoglobin' OR hemoglobin OR 'blood sugar' OR 'hemoglobin a1c'/exp OR 'hemoglobin a1c' OR 'hba1c'/exp OR hba1c OR a1c OR 'glycemic control'/exp OR 'glycemic control' OR 'glycaemia'/exp OR glycaemia OR 'glycemia'/exp OR glycemia) AND (intensive OR tight OR conventional) AND (diabetes) AND ('diabetic retinopathy' OR retin*) AND ('randomized controlled trial' OR random*) | 280 |
| ScienceDirect | Title, abstract, keywords: (glycemic OR glycemia OR glycaemia OR "blood glucose" OR HbA1c) AND (retinopathy) AND diabetes AND ("randomized controlled trial") | 6 |

**Supplement Table 2. The baseline of diabetic retinopathy condition**

| **Study** | **In the original classification** | | |  | **International Clinical DR Disease Severity Scale** | | |
| --- | --- | --- | --- | --- | --- | --- | --- |
|  | **DR conditions** | **Intensive Glycemia control** | **Conventional Glycemia control** | | **DR conditions** | **Intensive Glycemia control (%)** | **Conventional Glycemia control (%)** |
|  |  | **No. Group/Total (%)** | **No. Group/Total (%)** |  |  |  |  |
| **DCCT, 1993 Primary Prevention** | None | 348/348 (100) | 378/378 (100) |  | No apparent retinopathy | 100 | 100 |
| **DCCT, 1993 Secondary Prevention** | None | 263/363 (72.5) | 252/352 (71.6) |  | No apparent retinopathy | 72.5 | 71.6 |
|  | Microaneurysms | 67/363 (18.5) | 58/352 (16.5) |  | Mild NPDR | 18.5 | 16.5 |
|  | Mild NPDR | 18/363 (5.0) | 23/352 (6.5) |  | Moderate NPDR | 5.0 | 6.5 |
|  | Moderate NPDR | 15/363 (4.1) | 19/352 (5.4) |  | Severe NPDR and PDR | 4.1 | 5.4 |
| **VADT, 2014** | ETDRS |  |  |  |  |  |  |
|  | 10 | 128/433 (29.6) | 135/425 (31.8) |  | No apparent retinopathy | 29.6 | 31.8 |
|  | 20 | 93/433 (21.5) | 89/425 (20.9) |  | Mild NPDR | 21.5 | 20.9 |
|  | 35 | 108/433 (24.9) | 112/425 (26.4) |  | Moderate NPDR | 42.7 | 41.2 |
|  | 43 | 77/433 (17.8) | 63/425 (14.8) |  | Severe NPDR and PDR | 6.2 | 6.1 |
|  | 60 | 27/433 (6.2) | 26/425 (6.1) |  |  |  |  |
| **UKPDS 33, 1998** | None | 1747/2729 (64.0) | 728/1138 (64.0) |  | No apparent retinopathy | 64 | |
|  | Retinopathy | 982/2729 (36.0) | 410/1138 (36.0) |  | Mild NPDR | 24 | |
|  |  |  |  |  | Moderate NPDR | 10 | |
|  |  |  |  |  | Severe NPDR and PDR | 2.1 | |
| **ACCORD, 2010** | None | 729/1428 (51.1) | 721/1426 (50.6) |  | No apparent retinopathy | 51.1 | 50.6 |
|  | Mild NPDR | 241/1428 (16.9) | 277/1426 (19.4) |  | Mild NPDR | 16.9 | 19.4 |
|  | Moderate NPDR | 433/1428 (30.3) | 404/1426 (28.3) |  | Moderate NPDR | 31.0 | 28.3 |
|  | Severe NPDR | 5/1428 (0.4) | 5/1426 (0.4) |  | Severe NPDR and PDR | 1.1 | 1.7 |
|  | PDR | 10/1428 (0.7) | 19/1426 (1.3) |  |  |  |  |
| **AdRem, 2009** | ETDRS |  |  |  |  |  |  |
|  | 10 | 393/791 (49.7) | 416/811 (51.3) |  | No apparent retinopathy | 59.8 | 59.9 |
|  | 14 | 80/791 (10.1) | 70/811 (8.6) |  | Mild NPDR | 22.3 | 21.9 |
|  | 20 | 176/791 (22.3) | 178/811 (21.9) |  | Moderate NPDR | 14.8 | 13.8 |
|  | 35 | 71/791 (9.0) | 63/811 (7.8) |  | Severe NPDR and PDR | 3.1 | 4.3 |
|  | 43 | 46/791 (5.8) | 49/811 (6.0) |  |  |  |  |
|  | 47 | 14/791 (1.8) | 13/811 (1.6) |  |  |  |  |
|  | 53 | 4/791 (0.5) | 12/811 (1.5) |  |  |  |  |
|  | 61 | 6/791 (0.8) | 10/811 (1.2) |  |  |  |  |

**Supplement Table 3. Diabetic retinopathy outcomes at the end of studies**

| **Study** | **DR conditions** | **Intensive Glycemic control** | **Conventional Glycemic control** | **P-value** |
| --- | --- | --- | --- | --- |
|  |  | **No. Group/Total (%)** | **No. Group/Total (%)** |  |
| **DCCT, 1993 Primary Prevention** | New-onset DR | 23/348 (7) | 91/378 (24) | 0.04 |
| **DCCT, 1993 Secondary Prevention** | DR progression | 77/363 (21) | 143/352 (41) | 0.01 |
| **UKPDS 33, 1998** | Retinopathy two-step ETDRS grading progression |  |  |  |
|  | 0-3y | 282/1786 (16) | 114/743 (15) | 0.78 |
|  | 0-6y | 352/1531 (23) | 178/640 (28) | 0.017 |
|  | 0-9y | 363/1171 (31) | 172/459 (37) | 0.012 |
|  | 0-12y | 202/523 (39) | 95/195 (49) | 0.015 |
| **VADT, 2014** | New-onset DR | 54/128 (42) | 66/135 (49) | 0.27 |
|  | DR progression | 69/406 (17) | 88/399 (22) | 0.07 |
| **ACCORD, 2010** | Retinopathy three-step ETDRS grading progression | 104/1429 (7) | 149/1427 (10) | 0.03 |
| **AdRem, 2009** | Retinopathy two-step ETDRS grading progression | 88/791 (11) | 99/811 (12) | 0.12 |
